# Pre-reproductive Parental Enriching Experiences Influence Progeny’s Developmental Trajectories

**DOI:** 10.3389/fnbeh.2018.00254

**Published:** 2018-11-12

**Authors:** Debora Cutuli, Erica Berretta, Daniela Laricchiuta, Paola Caporali, Francesca Gelfo, Laura Petrosini

**Affiliations:** ^1^Department of Psychology, Faculty of Medicine and Psychology, Sapienza University of Rome, Rome, Italy; ^2^Fondazione Santa Lucia, Rome, Italy; ^3^Department of Human Sciences, Guglielmo Marconi University, Rome, Italy

**Keywords:** environmental enrichment, maternal care, motor behavior, cognition, BDNF, oxytocin, stress response, rats

## Abstract

While the positive effects of environmental enrichment (EE) applied after weaning, in adulthood, during aging, or even in the presence of brain damage have been widely described, the transgenerational effects of pre-reproductive EE have been less examined. And yet, this issue is remarkable given that parental environmental experience may imprint offspring’s phenotype over generations through many epigenetic processes. Interactions between individual and environment take place lifelong even before conception. In fact, the environment pre-reproductively experienced by the mother and/or the father exerts a substantial impact on neural development and motor and cognitive performances of the offspring, even if not directly exposed to social, cognitive, physical and/or motor enrichment. Furthermore, pre-reproductive parental enrichment exerts a transgenerational impact on coping response to stress as well as on the social behavior of the offspring. Among the effects of pre-reproductive parental EE, a potentiation of the maternal care and a decrease in global methylation levels in the frontal cortex and hippocampus of the progeny have been described. Finally, pre-reproductive EE modifies different pathways of neuromodulation in the brain of the offspring (involving brain-derived neurotrophic factor, oxytocin and glucocorticoid receptors). The present review highlights the importance of pre-reproductive parental enrichment in altering the performances not only of animals directly experiencing it, but also of their progeny, thus opening the way to new hypotheses on the inheritance mechanisms of behavioral traits.

## Introduction

Organisms adapt their physiology and behavior in response to environmental modifications. The influence of environmental experiences during lifespan is confined not only to the neurobehavioral profiles of the directly exposed individual, but it can also be evident in the next generations (Arai and Feig, 2011; Thayer and Kuzawa, 2011; Lim and Brunet, 2013). Growing evidence demonstrates that both ancestral and parental histories are able to affect the offspring depending on the valence of the environmental circumstances and the period of exposure to them, and that environmental influences may be passed on to the next generations through epigenetic (i.e., non-DNA sequence-based rather than mutational) modifications (Jirtle and Skinner, 2007; Caldji et al., 2011; Bohacek and Mansuy, 2015; Wang et al., 2017; Weaver et al., 2017). These studies shed new light on the Lamarckian theories of the inheritance of acquired traits that have been a matter of debate for over a century (Dröscher, 2015; Wang et al., 2017).

It is well-known that pre-natal/early post-natal as well as ancestral experience of environmental insults (e.g., undernutrition, stress) may result in modifications of the phenotype later in life (Shachar-Dadon et al., 2009; Franklin and Mansuy, 2010; Veenendaal et al., 2012; Xu et al., 2016; Ambeskovic et al., 2017). In the present review article we would address the effects of pre-reproductive enriching stimulations experienced by parents, such as the exposure to environmental enrichment (EE) or even exercise, on the behavioral and neurobiological phenotype of the offspring in rodents.

Environmental enrichment is an experimental paradigm apt to potentiate social, cognitive, and sensorimotor stimulations experienced by animals (Rosenzweig et al., 1964). By increasing environmental novelty and complexity, EE exerts potential therapeutic and neuroprotective effects as demonstrated by its efficacy in enhancing neural plasticity and delaying the progression and/or ameliorating the symptoms in the presence of brain injuries and diseases (Nithianantharajah and Hannan, 2006; Baroncelli et al., 2009; Nithianantharajah and Hannan, 2009; Petrosini et al., 2009; Cutuli et al., 2011; Simpson and Kelly, 2011; Sale et al., 2014; Mandolesi et al., 2017; Sampedro-Piquero and Begega, 2017; Gelfo et al., 2018). The broad beneficial effects of EE probably result from complex interactions among time window of exposure to EE, type of enrichment, and gender of enriched animals (Girbovan and Plamondon, 2013). On the other hand, scattered negative outcomes have also been reported, probably linked to the enhanced stress levels induced by the EE protocol (Schilling et al., 2004; Wood et al., 2011; Huzard et al., 2015; Mo et al., 2016).

Environmental enrichment as well as exercise may improve learning and memory, and enable neuroplasticity processes involving increased neurogenesis, possibly via neurotrophin-mediated mechanisms (Kempermann et al., 1997; van Praag et al., 1999; Olson et al., 2006; Bechara and Kelly, 2013; Livingston-Thomas et al., 2016). Notably, EE and exercise effects are difficult to disentangle, because most enrichment paradigms incorporate exercise elements. Anyway, many studies demonstrated that the motor component (i.e., voluntary running-wheel exercise) is the major neurogenic and neurotrophic stimulus in EE protocols in comparison to the cognitive and social components (i.e., complex environments comprised of inanimate objects with or without social interactions, but without running-wheel) (Kobilo et al., 2011; Mustroph et al., 2012; Bechara and Kelly, 2013; Grégoire et al., 2014).

In the last years, attention has been paid to the transfer of proactive effects of enriching experiences from parents to the progeny, and the number of researches on this issue is significantly increased over time (Arai and Feig, 2011; Girbovan and Plamondon, 2013; Sale et al., 2014; Taouk and Schulkin, 2016; Sale, 2018). By using different rat and mouse strains, schedules and protocols of physical, social and/or cognitive enrichment, and various behavioral tests, these studies demonstrated that parental positive manipulations are able to alter the neurodevelopmental trajectories of the progeny, likely to prepare the fetus to cope with a specific environment.

The effects of parental enrichment across generations have been mainly investigated by exposing mothers to different kinds of environmental stimulations during gestation and/or lactation (Sale et al., 2014).

A rising literature demonstrates the role of maternal (Dell and Rose, 1987; Arai et al., 2009; Leshem and Schulkin, 2012; Caporali et al., 2014, 2015; Cutuli et al., 2015, 2017, 2018) or paternal (Mashoodh et al., 2012; Mychasiuk et al., 2012; Dezsi et al., 2016; Short et al., 2017; Yeshurun et al., 2017) enrichment “before conception” (i.e., during the pre-reproductive period) in modifying the neurobiological and behavioral profile of the offspring. Since early nurturing experiences may influence brain plasticity and alter epigenome (Meaney, 2010; Weaver et al., 2017), part of these studies have also evaluated if the effects of pre-reproductive parental enrichment on offspring’s phenotype were mediated by modifications of maternal behavior (Mashoodh et al., 2012; Caporali et al., 2015; Cutuli et al., 2015, 2017, 2018; Short et al., 2017; Yeshurun et al., 2017).

To our knowledge, the literature on the effects of early EE by parents in humans is still scarce, and principally deals with fetuses’ and newborns’ stimulation. In fact, besides an accumulating evidence on the beneficial consequences of tactile (i.e., body massage) and auditory stimulation (i.e., exposure to maternal voice) on the neurobehavioral development of preterm infants (Guzzetta et al., 2009; Picciolini et al., 2014; Webb et al., 2015) or the effects of antenatal auditory stimulations (i.e., training with music and maternal talk to the fetus during pregnancy) on the reduction of autistic-like behaviors (Ruan et al., 2018), to date only one article has demonstrated the proactive influence of pre-reproductive enrichment represented by maternal educational achievement on buffering offspring’s stress sensitivity (Swartz et al., 2018).

In the following paragraphs, we will take into account the impact of pre-reproductive parental exposure to enriching experiences on maternal behavior and on physical, motor, cognitive and emotional features of the offspring in rodents. Furthermore, based on our studies and the few others present in literature, we will focus on the changes in a key neurotrophic factor, such as the brain-derived neurotrophic factor (BDNF), and in the oxytocinergic system following pre-reproductive parental housing in highly stimulating environments. Whenever possible, comparisons with the effects of parental enrichment during gestational and/or lactation periods (i.e., post-reproductive enrichment) will be also considered. Namely, in Table 1 for each study we defined the period of enrichment exposure and if the enrichment was maternal and/or paternal, social (if the enriched cages contained more individuals than in the control groups), cognitive (if the objects, toys and other materials inside the enriched cages were systematically changed, rearranged or renewed), physical (if the enriched cages were bigger than the standard ones and contained objects, toys, igloos, tunnels, nesting materials, etc.) and/or motor (with one or more running-wheels). It is evident that in literature different EE paradigms has been used, with little understanding of how differences in individual EE variables (such as social, cognitive, physical and/or motor ones) might impact on specific downstream biological mechanisms. Thus, given the importance of the motor component in EE (as above discussed), exercise only paradigms (by running-wheel, motor driven treadmill or swimming) were also taken into account as forms of parental enriching experiences. In addition, we described the effects of communal nesting, a condition in which parental responsibilities are shared by multiple individuals in a nest, as a source of social extra-stimulation in pups’ early life.

**Table 1:** Parental enrichment protocols.

Parental enrichment protocols					

A. Protocols of pre-reproductive parental enrichment					

Authors	Year	Animals	Kind of enrichment	Enrichment period	Kind of parental enrichment
Arai et al. (2009)	2009	Ras-grf k/o mice	Moderate social, cognitive, physical and motor enrichment (with running-wheel)	Pre-reproductive period	Maternal or paternal
Benito et al. (2018)	2018	C57Bl/6J mice	Cognitive, physical and motor enrichment (with running-wheels)	Pre-reproductive period	Paternal
Caporali et al. (2014)	2014	Wistar rats	Social, cognitive, physical and motor enrichment (with running-wheel)	Pre-reproductive period	Maternal
Caporali et al. (2015)	2015	Wistar rats	Social, cognitive, physical and motor enrichment (with running-wheel)	Pre-reproductive period	Maternal
Champagne and Meaney (2007)	2007	Long–Evans rats	Social, cognitive and physical enrichment (without running-wheel)	Pre-reproductive period	Maternal
Cutuli et al. (2015)	2015	Wistar rats	Social, cognitive, physical and motor enrichment (with running-wheels)	Pre-reproductive period	Maternal
Cutuli et al. (2017)	2017	Wistar rats	Social, cognitive, physical and motor enrichment (with running-wheel)	Pre-reproductive period	Maternal
Cutuli et al. (2018)	2018	Wistar rats	Social, cognitive, physical and motor enrichment (with running-wheel)	Pre-reproductive period	Maternal
Dezsi et al. (2016)	2016	Genetic Absence Epilepsy rats	Cognitive, physical and motor enrichment (with running-wheel)	Pre-reproductive period	Paternal
Mashoodh et al. (2012)	2012	BALB/c mice	Social, physical and motor enrichment (with running-wheel)	Pre-reproductive period	Paternal
Short et al. (2017)	2017	C57Bl/6 mice	Motor enrichment (by running-wheel)	Pre-reproductive period	Paternal
Yeshurun et al. (2017)	2017	C57Bl/6J mice	Cognitive and physical enrichment (without running-wheel)	Pre-reproductive period	Paternal

**B. Protocols of parental enrichment during pregnancy**					

**Authors**	**Year**	**Animals**	**Kind of enrichment**	**Enrichment period**	**Kind of parental enrichment**

Cymerblit-Sabba et al. (2013)	2013	Wistar rats	Social, cognitive, physical and motor enrichment with running-wheel	Pregnancy	Maternal
Gomes Da Silva et al. (2016)	2016	Wistar rats	Motor enrichment (by motor driven treadmill)	Pregnancy	Maternal
Herring et al. (2012)	2012	TgCRND8 mice	Motor enrichment (by running-wheel)	Pregnancy	Maternal
Kiyono et al. (1985)	1985	Fischer rats	Social, cognitive and physical enrichment (without running-wheel)	Pregnancy	Maternal
Koo et al. (2003)	2003	Sprague–Dawley rats	Social, cognitive, physical and motor enrichment (with running-wheel)	Pregnancy	Maternal
Lee et al. (2006)	2006	Sprague–Dawley rats	Motor enrichment (by swimming)	Pregnancy	Maternal
McKim and Thompson (1975)	1975	Sprague–Dawley rats	Physical and social enrichment	Pregnancy	Maternal
Park et al. (2013)	2013	C57Bl/6J mice	Motor enrichment (by motor driven treadmill)	Pregnancy	Maternal
Parnpiansil et al. (2003)	2003	Sprague–Dawley rats	Motor enrichment (by motor driven treadmill)	Pregnancy	Maternal
Rosenfeld and Weller (2012)	2012	WKY and Wistar rats	Cognitive, physical and motor enrichment (with running-wheel)	Pregnancy	Maternal

**C. Protocols of parental enrichment during pregnancy and lactation or only during lactation**					

**Authors**	**Year**	**Animals**	**Kind of enrichment**	**Enrichment period**	**Kind of parental enrichment**

Bick-Sander et al. (2006)	2006	C57Bl/6 mice	Motor enrichment (by running-wheel)	Pregnancy and lactation	Maternal
Branchi et al. (2010)	2010	CD-1 mice	Social and physical enrichment (without running-wheel)	Pregnancy and lactation	Maternal
Cancedda et al. (2004)	2004	C57Bl/6J mice, 148 CRE-LacZ transgenic mice	Social, cognitive and physical enrichment (without running-wheel)	Pregnancy and lactation	Maternal
Durán-Carabali et al. (2018)	2018	Wistar rats	Social, cognitive, physical and motor enrichment (with running-wheel)	Pregnancy and lactation	Maternal
Heiderstadt et al. (2014)	2014	C57BL/6J, DBA/2J and 129x1/SvJ mice	Social enrichment (without running-wheel)	Lactation	Maternal
Sale et al. (2004)	2004	C57Bl/6J mice	Social, cognitive, physical and motor enrichment (with running-wheel)	Pregnancy and lactation	Maternal
Sparling et al. (2010)	2010	Long–Evans rats	Physical and social enrichment (without running-wheel)	Pregnancy and lactation	Maternal
Welberg et al. (2006)	2006	Long–Evans rats	Moderate physical enrichment (without running-wheel)	Pregnancy and lactation	Maternal

**D. Mixed protocols of parental enrichment**					

**Authors**	**Year**	**Animals**	**Kind of enrichment**	**Enrichment period**	**Kind of parental enrichment**

Bechard and Lewis (2016)	2016	Peromyscus maniculatus (deer mice)	Social, cognitive, physical and motor enrichment (with running-wheel)	Pre-reproductive and pregnancy periods	Maternal and paternal
Connors et al. (2015)	2015	Sprague–Dawley rats	Physical and cognitive enrichment (without running-wheel)	Pre-reproductive, pregnancy and lactation periods	Maternal
Curley et al. (2009)	2009	Balb/c mice	Social and physical enrichment (without running-wheel)	Lactation (F0), pre-reproductive period (F1)	Maternal
Leshem and Schulkin (2012)	2012	Sprague–Dawley rats	Social, cognitive, physical and motor enrichment (with running-wheels)	Pre-reproductive period (F0)/post-weaning (F0)	Maternal
Maruoka et al. (2009)	2009	C57Bl/6J mice	Cognitive, physical and motor enrichment (with running-wheel)	Pre-reproductive and pregnancy periods	Maternal
Mychasiuk et al. (2012)	2012	Long–Evans rats	Social, cognitive and physical enrichment (without running-wheel)	Pre-reproductive and pregnancy periods (mothers), pre-reproductive period (fathers)	Maternal or paternal
Zuena et al. (2016)	2016	Wistar rats	Social, cognitive, physical and motor enrichment (with running-wheel)	Pre-reproductive and pregnancy periods	Maternal


*For each study the first author’s name, year of publication, kind of animals used, kind of enrichment (social, cognitive, physical and/or motor), period of enrichment exposure (during pre-reproductive, pregnancy and/or lactation periods) and kind of parental enrichment (maternal and/or paternal) are reported. In each sub-section (A–D), the studies are reported in alphabetical order.*

## Impact of Differently Timed Parental Enrichment on Maternal Behavior

In mammals, the parental care ensures that the offspring effectively will survive until the reproductive age, thus transmitting the genetic information across generations. The maternal care in rodents is made up of a “*constellation*” of behaviors of preparation for the arrival of the newborns as well as their nurturing and protection (Kristal, 2009).

The literature especially reports maternal behavior alterations following various protocols of enrichment during gestational and/or lactation periods, while fewer studies are available on the effects of pre-reproductive parental enrichment.

### Effects of Maternal Enrichment During Pregnancy and/or Lactation

Maternal exposure to EE during pregnancy and lactation increases maternal licking behavior, passive nursing and presence in the nest (Cancedda et al., 2004; Sale et al., 2004; Durán-Carabali et al., 2018).

Lactating rat dams enriched only during gestation display more pup-directed behaviors than control-reared rats during the first and third lactation weeks, but decrease the nighttime frequency of presence in the nest and licking/grooming (LG) and arched-back nursing (ABN) during the third lactation week (Rosenfeld and Weller, 2012).

Mother rats enriched before and during gestation show heightened LG behavior and reduced pup nursing (Zuena et al., 2016).

Maternal enrichment during pre-reproductive, gestational and lactation phases induces an increase in ABN, but a reduction in the time spent in the nest and nursing (Connors et al., 2015).

There are also studies in which parental enrichment does not affect (Bechard and Lewis, 2016) or even worsen the maternal behavior (Welberg et al., 2006). In fact, the study by Bechard and Lewis (2016) used a biparental EE protocol in deer mice during pre-reproductive and gestational phases, and found no differences in maternal behavior of enriched mothers. The study by Welberg et al. (2006) used a moderate EE protocol in rats during pregnancy and lactation, and found no differences in pup licking, but reduced nursing episodes and presence in the nest.

Taken together these data, even somewhat contrasting, seem to account for a modulatory effect of environmental conditions on maternal care that reflects the different EE protocols used or the timing of enrichment exposure.

The more effective EE protocols for increasing maternal care are the ones providing at least social, cognitive and physical stimulations during pregnancy and lactation (Cancedda et al., 2004; Sale et al., 2004; Durán-Carabali et al., 2018). Moreover, when EE protocol involves the transition from the enriched gestational environment to the standard environment, the increase in some pup-directed behaviors may be a substitute to object exploration activity, given the decreased space and stimuli (Rosenfeld and Weller, 2012). A similar explanation can be valid also if females enriched in groups before and during gestation are individually housed in enriched cages near parturition (Zuena et al., 2016).

A variable conserved across dams housed in EE is the reduction in the time/frequency in contact with the nest. It may be attributed to the increased physical space of the enriched cage, when the EE protocol is applied at least during gestation and lactation (Welberg et al., 2006; Connors et al., 2015), given the enriched housing conditions reproduce the natural situation in which the dam is given more space to explore and thus more chance to leave the nest. On the contrary, the reduced maternal contact with the nest can be a sign of distress induced by the transition from the enriched gestational environment to the standard environment; this transition may have an aversive meaning leading to a premature withdrawal of EE dams from their pups (i.e., spending less time in the nest and performing less LG and lactation in the third post-natal week) (Rosenfeld and Weller, 2012). This behavioral pattern seems analogous to the early weaning manipulation, where the pups are separated from their mothers in the third post-natal week and become more anxious and stressful (Ito et al., 2006; Kikusui et al., 2006).

### Effects of Pre-reproductive Parental Enrichment

Few researches have investigated the effects of pre-conceptional parental enrichment on maternal care to date. In particular, when rearing their own offspring under standard conditions, communally reared females are reported to perform enhanced levels of *post-partum* care in comparison to standard-reared females, and their offspring show increased frequency of nursing (Curley et al., 2009).

Post-weaning exposure to EE of low LG offspring enhances LG behavior and oxytocin receptor binding across generations (Champagne and Meaney, 2007).

Recently, we have demonstrated that pre-reproductive maternal EE induces maternal care modifications consisting of higher levels of licking, ABN, and nest building activities, and a faster retrieving after maternal male intruder encounters (Cutuli et al., 2015, 2017, 2018). These effects of the pre-reproductive exposure of mothers to EE are accompanied by heightened levels of BDNF in the frontal cortex at pups’ weaning (Caporali et al., 2015; Cutuli et al., 2015).

Paternal environmental experiences can modify offspring’s phenotype even in the absence of paternal care. Unfortunately, only few studies addressed the role of mothers in the transmission of paternal effects by analyzing maternal care. Namely, standard-reared female mice mated with male mice enriched during the pre-reproductive period show increased frequency of pup nursing and licking during the first *post-partum* week. Such behavioral modifications are associated with gene expression modifications (i.e., higher levels of BDNF mRNA and lower levels of MeCP2 mRNA) in the hypothalamus of dams (Mashoodh et al., 2012). The effects of paternal enrichment via maternal investment could be the consequence of inherited paternal epigenetic variations that lead to variations in the level of maternal care requested by the offspring. In fact, pups provide distal cues (e.g., sight, sound, tactile contact) for the mother, thus stimulating her contact with them. Interestingly, previous studies demonstrated that locomotion, ultrasonic vocalization and suckling ability are influenced by paternal genes (Curley et al., 2004; Plagge et al., 2004; Swaney, 2011). It can be speculated that the modulation of maternal behavior in standard-reared females mated with enriched males can be linked to a more demanding behavioral pattern of the pups.

Conversely, more recent studies did not find any effect of paternal enrichment on the maternal behavior (Short et al., 2017; Yeshurun et al., 2017).

Overall, the majority of the studies reported in this section seems to indicate that different pre-reproductive parental enriching experiences can potentiate the maternal care. Anyway, divergent data are emerged about the effects of pre-reproductive paternal enrichment on maternal behavior, possibly due to methodological reasons. In fact, in the study by Mashoodh et al. (2012) the fathers received increased social, physical and motor stimulations during their entire lifetime before breeding, and they were compared to male mice reared in isolated conditions. Differently, in the studies by Short and colleagues and Yeshurun and colleagues the fathers were exposed to only motor or only cognitive and physical enrichment and just in adulthood. Furthermore, in the study by Short et al. (2017) runner fathers and controls were both single-housed, while in the study by Yeshurun et al. (2017) enriched fathers and controls were both socially reared (four mice per cage).

It seems that the more is complete and long-lasting the set of stimulations provided to the fathers, more intense is the potentiating effects on the maternal behavior.

Furthermore, the use of socially isolated males (instead of standard-reared males) in the study by Mashoodh et al. (2012) could have contributed to shift the behavioral and neurobiological phenotypes of mates to extremes, thus influencing the maternal investment. Anyway, Mashoodh et al. (2012) showed that maternal investment is only partially dependent on the social and environmental experience of the mate (since environmentally induced anxiety levels of males did not fully predict the frequency of maternal nursing).

As evident, the implications of environmental factors on the complex relationship among paternal, maternal and offspring phenotypes still represent a crucial challenge in the study of the mechanisms driving paternal effects.

## Impact of Differently Timed Parental Enrichment on the Offspring’s Phenotype

### Effects of Pre-reproductive vs. Post-reproductive Parental Enrichment on Physical Development and Motor Behavior

A proper assessment of the transgenerational effects of parental experiences on offspring developmental trajectories can be achieved by examining the maturation of motor behaviors in rodents. This represents a useful tool to carefully assess early post-natal neurodevelopment, because the appearance of sensorimotor reflexes and motor skills typically follows a definite timing during the first 3 weeks after birth (De Souza et al., 2004).

Unfortunately, few studies paid specific attention to the transgenerational effects of EE on offspring’s motor development and even fewer ones used the pre-reproductive EE paradigm, although it shows the possibility to distinguish pre- from post-natal effects of EE, thus allowing the study of Lamarckian inheritance.

To this aim, we performed a series of studies to examine the hypothesis that pre-reproductive maternal EE could affect progeny’s phenotype, by rearing female Wistar rats in an enriched environment from weaning until mating (Caporali et al., 2014, 2015; Cutuli et al., 2015, 2017, 2018). To our knowledge, the study by Caporali and colleagues is the only available research analyzing the transgenerational effects of pre-reproductive maternal EE on offspring’s development by using a battery of tests examining the acquisition of several developmental milestones in the physical and sensorimotor development (Caporali et al., 2014).

This pre-reproductive maternal EE did not affect litter characteristics, such as litter size and male/female ratio, thus suggesting that this experience does not influence the reproductive ability or the pregnancy of female rats (Caporali et al., 2014; Cutuli et al., 2015, 2017). Interestingly, similar results have been reported following differently timed maternal EE protocols (Welberg et al., 2006; Bechard and Lewis, 2016; Zuena et al., 2016) as well as following pre-reproductive paternal enrichment (Mashoodh et al., 2012; Mychasiuk et al., 2012; Yeshurun et al., 2017).

We found that pre-reproductive maternal enrichment affects offspring’s body weight only at birth, with enriched mothers’ offspring weight less than controls (Cutuli et al., 2015, 2017), similarly to their own enriched mother. As suggested by Sparling et al. (2010), this finding is consistent with researches demonstrating that enriched females are leaner (Pham et al., 1999; Larsson et al., 2002; Olsson and Dahlborn, 2002; Moncek et al., 2004; Brillaud et al., 2005) and maintain their weight stable over time (Brillaud et al., 2005). However, even opposing results have been collected. In fact, when female rats are exposed to a social colony designed to provide enhanced physical and social stimulations, during pregnancy and lactation, they give birth to heavier offspring (Sparling et al., 2010). Given higher weight of the offspring at birth is retained a good predictor of developmental success (Byrd and Weitzman, 1994; Prathanee et al., 2009), the authors conclude that heavier colony pups would have got a physiological advantage compared to their control mates (Sparling et al., 2010). No differences in the body weight have been reported in adulthood, even following different schedules of maternal enrichment (Welberg et al., 2006; Maruoka et al., 2009; Zuena et al., 2016). The pre-reproductive paternal exposure to enriched social, cognitive and physical stimulations (regardless running-wheel) does not affect body weight at birth, but it does predict male offspring’s weight at adulthood, resulting in an increased weight gain (Mashoodh et al., 2012) that persists until the second generation (Yeshurun et al., 2017).

Overall, it seems that parental environmental experiences affect the body weight of the progeny. Namely, enriched mothers influence progeny’s body weight only at birth, in a way ambiguously linked to different EE conditions, whereas enriched fathers influence offspring’s body weight at adulthood. Thus, it is possible to speculate that both maternal and paternal influences are driven by pre-natal metabolic programming, even if we cannot exclude that a specific maternal behavior in the pre-weaning phase may account for the particular fitness outcome observed in the progeny of enriched fathers (Mashoodh et al., 2012).

The pre-reproductive maternal EE alters the motor development of the progeny, leading to an earlier acquisition of those abilities that require complex sequencing and coordination of the motor output (Caporali et al., 2014). Furthermore, maternal enrichment does not influence the postural development or affect the appearance of dynamic sensorimotor reflexes (negative geotaxis, cliff avoidance, and vestibular drop). Interestingly, similar results on negative geotaxis acquisition were obtained by Mychasiuk et al. (2012) evaluating the effects of pre-reproductive paternal enrichment. Nevertheless, the same authors found that the offspring of females enriched prior to and during pregnancy exhibit a reduction in time required to show negative geotaxis, but no EE effects were recorded on the day of the appearance. Thus, one possibility is that maternal EE, only if gestational, affects the time required to show negative geotaxis as days go by. A similar effect on negative geotaxis performance has been reported also by combining pre- and post-natal EE: the maternal exposure to social, cognitive, physical and motor enrichment occurring during pregnancy and lactation is able to promote a better reflex performance (i.e., reduced latency) counteracting the neurobehavioral delay induced by neonatal hypoxia ischemia, a common neurological complication occurring in preterm infants (Durán-Carabali et al., 2018). Given some researches demonstrated that post-natal EE exposure positively influences negative geotaxis development (Kiss et al., 2013; Schuch et al., 2016), it is possible that direct exposure to EE of fetus first and pup later leads to a better performance, without affecting the day of the appearance.

The open field test (OF) is one of the most frequently used method to assay locomotor behavior in rodents and is sensitive to EE-induced exploratory modifications. The analysis of the effects of pre-reproductive parental enrichment on explorative activity of the progeny provided conflicting results. In fact, increased OF activity levels have been reported in young male offspring of pre-reproductively enriched fathers (Mychasiuk et al., 2012), while adult male offspring of pre-reproductively enriched mothers exhibit activity levels similar to controls (Cutuli et al., 2015). Opposite findings were also obtained with differently timed maternal EE protocols. Mychasiuk et al. (2012) found increased OF activity levels in young male and female offspring of mothers enriched prior to and during pregnancy. Even cross-fostered pups of female rats exposed to EE during pregnancy show increased locomotion in the OF (McKim and Thompson, 1975). Anyway, reduced OF activity levels have been reported in young and adult female offspring in other experimental conditions (Maruoka et al., 2009; Rosenfeld and Weller, 2012). The conflicting findings now described, along with the well-known influence exerted by several factors on OF activity levels [for a review see (Simpson and Kelly, 2011)], make this issue worthy of further investigation.

In conclusion, the exposure of rodents to EE during the pre-reproductive phase significantly shapes the neurodevelopment of progeny, by accelerating the acquisition of motor abilities (Caporali et al., 2014). No significant effects have been described on litter features (Mashoodh et al., 2012; Mychasiuk et al., 2012; Caporali et al., 2014; Cutuli et al., 2015, 2017; Yeshurun et al., 2017), while contrasting findings, probably linked to different EE protocols and experimental parameters, have been reported on body weight and OF activity levels.

### Effects of Pre-reproductive vs. Post-reproductive Parental Enrichment on Cognition and Anxiety

#### Effects of Maternal Enrichment During Pregnancy and/or Lactation

In rodents the effects of parental enrichment on the offspring’s cognitive performances and anxiety levels have been primarily investigated by exposing mothers to complex stimulations during pregnancy and/or lactation, and by using different kinds of behavioral tasks and biochemical correlates. Namely, the exposure of mothers to different kinds of EE paradigms during pregnancy facilitates learning and memory abilities (Kiyono et al., 1985; Koo et al., 2003), and increases synaptic plasticity and hippocampal neurogenesis in the progeny (Koo et al., 2003). In addition, the maternal EE exposure during pregnancy reduces attentional performance (Cymerblit-Sabba et al., 2013), and controversially affects anxiety levels of the offspring (Maruoka et al., 2009; Rosenfeld and Weller, 2012; Cymerblit-Sabba et al., 2013).

Maternal exposure to EE during pregnancy and lactation improves spatial memory performances in the Morris water maze, reduces anxiety, and prevents the hippocampal tissue loss after neonatal hypoxia ischemia (Sparling et al., 2010; Durán-Carabali et al., 2018).

Also the exposure of mothers to exercise only paradigms during pregnancy (Parnpiansil et al., 2003; Lee et al., 2006; Herring et al., 2012) or during pregnancy and lactation (Bick-Sander et al., 2006) is able to improve learning and memory abilities, protect from neurodegeneration, improve brain plasticity and enhance hippocampal neurogenesis in the offspring.

The maternal exposure to EE before and during gestation affects offspring’s development trajectories by modifying cognitive and emotional outcomes in a sex-specific manner with improved learning ability only in females and increased anxiety mainly in males (Connors et al., 2015; Zuena et al., 2016).

Lastly, data resulting from post-natal social enrichment obtained by communal nesting are variable and still difficult to reconcile across studies. For example, a study by Heiderstadt et al. (2014) does not find any difference induced by communal nesting in learning or anxiety tests in the adult offspring. Conversely, in a study by Curley et al. (2009) communal nesting reduces anxiety levels and modifies oxytocin and vasopressin receptor densities in the adult offspring. These results were obtained by using different mice strains [i.e., C57BL/6J, DBA/2J and 129x1/SvJ mice (Heiderstadt et al., 2014) vs. Balb/c mice (Curley et al., 2009)], and suggest the importance of considering strain-specific effects in interpreting the long-term developmental effects of early social enrichment. In fact, communal nesting may be particularly effective and beneficial when using the Balb/c strain, since these mice display a more stress vulnerable phenotype [e.g., elevated stress responses and behavioral inhibition accompanied by reduced hippocampal glucocorticoid receptor expression and increased corticosterone (CORT) levels] (Brinks et al., 2007; Brodkin, 2007).

#### Effects of Pre-reproductive Parental Enrichment

The few studies available on the effects of pre-conceptional parental enrichment on the cognition and emotional response of the offspring generally indicate an amelioration of memory performances and a reduced anxiety. In fact, Arai et al. (2009) found rescued contextual fear conditioning memory and enhanced long-term potentiation not only in mice directly exposed to 2-weeks of EE when juveniles, but also in their offspring that never experienced EE. Benefits of EE across generations are associated with a signaling cascade in the CA1 hippocampal region and pass on through the mother (Arai et al., 2009). In rats, intergenerational effects of females’ exposure to EE from weaning to reproductive age include improvements in cognitive performances as well as increased hippocampal BDNF levels in their male offspring, without changes in neurogenesis or reelin levels (Cutuli et al., 2015). A similar protocol of pre-reproductive maternal enrichment does not influence avoidance learning, and induces sex-dependent effects in female offspring by reducing anxiety and improving habituation to acoustic startle (Leshem and Schulkin, 2012).

Low LG female offspring housed under enriched conditions display increased LG behavior and higher levels of exploration in comparison to standard housed low LG females (Champagne and Meaney, 2007). These variations are also passed to the progeny.

In addition, communal nesting is able to increase maternal care and reduce anxiety across generations (Curley et al., 2009).

As for pre-reproductive paternal enrichment experiences, the existing studies mainly addressed the emotional responses of the offspring. Namely, a very recent study by Benito et al. (2018) demonstrated that pre-conceptional exposure of adult male mice to EE enhances LTP and induces a subtle memory improvement in the next generation, and that this phenotype is mediated by changes in the RNA composition in the sperm of the enriched fathers, especially through the upregulation of microRNA 212 and 132.

In a genetic rat model of absence epilepsy, early enrichment of fathers from weaning to breeding induces anti-epileptogenic and anxiolytic effects that were heritable across generations (Dezsi et al., 2016). These findings are in line with the anxiolytic effects of pre-reproductive paternal exercise. In fact, the male offspring of runner fathers show reduced anxiety levels and more robust fear extinction memory associated with alterations in the levels of small non-coding RNAs in sperm (Short et al., 2017). Conversely, it has been recently demonstrated that when the motor enrichment component is lacking, no differences in anxiety are found following pre-reproductive paternal enrichment (Yeshurun et al., 2017).

In conclusion, it seems that pre-reproductive paternal housing conditions which include an overt motor enrichment by running-wheel presence are able to induce anxiolytic effects.

With regard to indirect effects of parental enriching experiences on the pups’ behavioral trajectories through the modulation of maternal care, the few data currently available are still little explicative, since maternal behavior has not been systematically investigated. However, it can be noted that when an enhancement in maternal care is evident following pre-reproductive maternal enriching experiences, the offspring’s phenotype can be characterized by enhanced maternal care and/or reduced anxiety (Champagne and Meaney, 2007; Curley et al., 2009) or by improved cognitive performances (Cutuli et al., 2015).

As for paternal enrichment, it seems that a clear relationship between maternal care and offspring’s behavior is still not well-definite. In fact, studies reporting behavioral modifications in the offspring of not-enriched females mated with males enriched during the pre-reproductive period fail to report maternal care modifications, thus suggesting that the progeny could be differently influenced by the pre-reproductive paternal experiences according to the different environmental manipulations used (motor enrichment vs. cognitive and physical enrichment), regardless maternal investment (Short et al., 2017; Yeshurun et al., 2017). Interestingly, enriching fathers preconceptionally significantly reduced global methylation levels in the frontal cortex and hippocampus of the developing offspring (Mychasiuk et al., 2012). And, other studies found modifications of RNA expression in the sperm (Short et al., 2017; Benito et al., 2018). As sperm development in rodents occurs continuously, as in humans, it can be speculated that pre-reproductive paternal enrichment experiences are able to alter gene expression in the sperm of sires, thus providing a means for the transmission of epigenetic change to the progeny.

### Effects of Pre-reproductive vs. Post-reproductive Parental Enrichment on Social Behavior

Social interactions are essential for survival and proper neural and behavioral development. After weaning, playful interactions with peers allow the acquisition of social and cognitive competence. Social play behavior is a highly rewarding activity in humans and animals, as it can instill a sense of well-being and pleasure, motivates approach behaviors toward a specific social stimulus, and finally elicits associative learning in order to attribute salience to socially related cues (Trezza et al., 2011; Vanderschuren et al., 2016). Interestingly, some studies indicate that EE may influence the social behavior in rodents. Namely, the exposure of pups to EE in the post-weaning period (Morley-Fletcher et al., 2003; Leshem and Schulkin, 2012) increases social interaction at adulthood and adolescence, respectively. Also, maternal exposure to EE before and during gestation enhances social behavior in the adolescent offspring with contrasting results depending on sex [i.e., higher social contact duration in the female offspring (Connors et al., 2015), and higher social play behavior in the male offspring (Zuena et al., 2016)].

As for pre-reproductive maternal EE, two studies demonstrated that it reduces social interaction in males, but not in females either in adult and adolescent offspring (Leshem and Schulkin, 2012; Cutuli et al., 2018). Similar effects are found in the adult offspring of pre-reproductively stressed females (Leshem and Schulkin, 2012), and are in line with the generally increased vulnerability of male rats to developmental disruption of social behavior by gestational ethanol (Mooney and Varlinskaya, 2011). The reduction in social play, and in particular in play solicitation (i.e., Pouncing) found in the enriched dams’ male adolescent offspring by Cutuli et al. (2018), is probably linked to a less rewarding value of play initiation (Vanderschuren et al., 1997) or to not clarified neurohormonal modifications.

Nevertheless, pre-reproductive maternal EE does not induce any difference in sociability as assessed in the three-chamber sociability test (Cutuli et al., 2015), in which social interaction is prevented and the animal has to choose between a compartment containing a juvenile conspecific or an empty compartment.

### Effects of Pre-reproductive vs. Post-reproductive Parental Enrichment on Stress Response

Environmental enrichment has been considered as prevention/intervention strategy to counteract the negative consequences of stress (McCreary and Metz, 2016).

The ability to cope with environmental challenges is essential to be successfully adapted. To face acute and chronic stressors, the sympathetic system and the hypothalamic-pituitary-adrenal (HPA) axis guarantee the recruitment of necessary resources, the inhibition of non-necessary ones, and fine feedback mechanisms for homeostasis. Glucocorticoids (GCs) secreted from adrenal glands act peripherally and in the brain through their binding with mineralcorticoid (MR) and glucocorticoid (GR) receptors. GR are recruited when GCs levels are elevated, as in a stressful condition, and are involved in negative feedback on HPA axis (De Kloet et al., 2005; Herman et al., 2016; Mifsud and Reul, 2018).

Genetic background (Kundakovic et al., 2013; Andolina et al., 2015; Di Segni et al., 2016) and parental environment (Meaney, 2001) shape individual differences in the ability to cope with stress and in the susceptibility of its negative consequences (Belsky and Pluess, 2009; Karatsoreos and McEwen, 2011; Daskalakis et al., 2013; Boersma and Tamashiro, 2015).

Parents have a pivotal role in programming the offspring’s HPA axis functioning as widely demonstrated in rodents (Meaney, 2001; Enthoven et al., 2010), and more controversially in non-human primates (Sanchez, 2006) and humans (Tollenaar et al., 2011).

Parental stress has been associated with offspring’s greater risk of psychopathology (Glover, 2011), higher glucocorticoid sensitivity (Lehrner et al., 2014), immunological alterations (Laviola et al., 2004), enhanced neuronal activity (Bielas et al., 2014) and altered DNA methylation (Mulligan et al., 2012; Essex et al., 2013). Recently, epigenetic inheritance is assigned a captivating role in the intergenerational outcomes (Franklin et al., 2010), stressing the standing of “epigenetics prior to the birth” (Lo and Zhou, 2014).

Compelling evidence from animal models highlights the effectiveness of the EE in preventing, rescuing or normalizing the negative outcomes of stress (McCreary and Metz, 2016). In rodents post-weaning EE normalizes basal immune parameters altered by pre-natal stress (Laviola et al., 2004), reverses the negative effects of pre-natal stress on HPA axis reactivity and play behavior (Morley-Fletcher et al., 2003), attenuates CORT levels after restraint stress (Sztainberg et al., 2010) and exerts enduring effects on CORT daily pattern and levels in the response to a novel environment (Peña et al., 2009). Similar findings were also recently found in zebrafish (*Danio rerio*) (Marcon et al., 2018).

In rats long-lasting moderate maternal EE during pregnancy, and together with pups during lactation until weaning, modifies the response of the female offspring to a chronic stress (Welberg et al., 2006). Whereas chronically stressed females show increased basal CORT and reduced adrenocorticotropic (ACTH) levels in response to stress, no such effects neither on basal CORT nor in ACTH acute release are found in chronically stressed offspring exposed to early EE.

Moreover, the positive effects of EE on stress response are reported in different strains of mice. Branchi et al. (2010) research with outbred CD-1, Swiss-derived strain (ICR) of mice clearly demonstrates that social enrichment by communal nesting that provides maternal caregiving from three different mothers and higher peer interaction is able to modify the offspring’s behaviors and neuroendocrine response to stress, by reducing anhedonia and decreasing CORT levels in response to social stress, enhancing time spent in immobility at the forced swim test (FST) and finally dampening the response to 3-weeks fluoxetine treatment. Also, in Balb/c mice, an inbred strain known for its high anxiety-like behavior, social enrichment by communal nesting reduces offspring’s stress response when exposed to a novel environment (Curley et al., 2009).

Remarkably, the enhancement of stimulations provided by EE produces changes that propagate across generations influencing descendant’s future responses to chronic and acute stress (Taouk and Schulkin, 2016) and promoting resilience even at the germline level (Gapp et al., 2016). The authors (Gapp et al., 2016) evidenced the role of both negative and positive environmental manipulations (i.e., maternal stress and unpredictable maternal separation, and exposure to EE, respectively) in influencing potential epigenome trajectories across generations. In particular, they proved that the detrimental effects of early stress on male offspring coping behaviors (F1) are related to a significant increase in GR expression in hippocampus and showed the propagation of the stress-related negative outcomes up until the subsequent generation (F2). The intergenerational transmission of phenotype is associated with a decreased methylation in exons 1–7 of the GR gene both in the F2 offspring and in the F1 sperm. In addition, the authors disclosed that EE is effective in preventing the transgenerational effects of stress on F2. The GR gene hypomethylation found in the sperm of early-stressed males was in fact rescued by EE. A very recent study also emphasizes the role of specific sperm microRNAs in mediating the effects of paternal pre-conceptional EE (Benito et al., 2018).

Pre-reproductive EE can modify the effect of social isolation, in rats. The offspring born to standardly reared mothers and subjected to social isolation from weaning to adolescence show greater GR expression in amygdala compared to not-isolated controls, whereas socially isolated offspring born to pre-reproductively enriched mothers do not show any difference in GR expression when compared to the respective not-isolated controls. Furthermore, a blunted amygdaloid c-Fos immunoreactivity in response to the FST is evident in the offspring of pre-reproductively enriched mothers in comparison to the offspring of standardly reared dams (Cutuli et al., 2017).

Recently, the transgenerational effects of pre-conceptional paternal EE on despair behaviors and neuroendocrine phenotypes of the adult offspring in mice has been investigated (Yeshurun et al., 2017). EE effects skip the F1 generation and are sex-specific. While pre-conceptional paternal EE has no effect on despair behaviors of F1 male and female offspring neither on basal ACTH or CORT levels in response to FST, modifies F2 females’ behavioral despair and CORT response in the FST. The transgenerational effect of paternal EE is not mediated by paternally induced changes in maternal care since no differences in maternal behaviors between EE and standardly reared male paired mothers.

Leshem and Schulkin (2012) showed that pre-reproductive maternal enrichment ameliorates the anxiogenic effect induced by pre-reproductive stress on offspring. The above-mentioned study also demonstrates stress-like effects of EE.

Environmental enrichment and stress indeed activate similar circuitries and involve HPA axis activity and changes in GR expression and functionality (Larsson et al., 2002; Moncek et al., 2004).

Several theoretical models have been proposed to account for the beneficial or contrasting effects of EE on stress response. Crofton et al. (2015) had addressed the issue in the inoculation stress hypothesis framework: the enhanced and complex stimulation provided by EE would represent a continuous mild form of stress that inoculates animals to subsequent challenges, as a controlled exposure to an harmless vaccine would protect against future encounter of the disease. Besides, the transition from eustress to detrimental stress follows the non-linear inverted U-shaped dose–response curve for which optimal levels of functionality are obtained at the moderate CORT expression while very high (e.g., overstimulation) or very low (e.g., understimulation) levels can exert similar deleterious effects on the individual (Sapolsky, 2015).

Considering conflicting transgenerational EE effects, the degree of fitness between the environmental challenges experienced by the offspring and the environmental demands for which parents and grandparents have equipped future generations to cope with has to be keep in mind (Marshall and Uller, 2007; Daskalakis et al., 2012; Nederhof and Schmidt, 2012; Prizak et al., 2014).

Finally, transgenerational inheritance could interact with ingrained individual differences to determine the phenotypic scenarios “for better or for worse” (Belsky et al., 2009).

## Impact of Differently Timed Parental Enrichment on BDNF

Neurotrophins constitute a protein family whose components show analogous structure and exert an essential action on the development and function of the neurons. They regulate cell proliferation and differentiation, growth and readjustment of axons and dendrites, and plastic changes involved in synaptic function (Park and Poo, 2013). In particular, BDNF is retained a key-player in the translation of the experience in neural structure and function modifications and a trigger and mediator of synaptic plasticity (Cowansage et al., 2010; Bekinschtein et al., 2011; Aarse et al., 2016). The direct effects of EE on brain BDNF levels have been demonstrated in a large number of animal studies (Angelucci et al., 2009; Gelfo et al., 2011; Mosaferi et al., 2015; Novkovic et al., 2015).

Recently, it has been evaluated if the known EE effects on BDNF-mediated brain plasticity could be transgenerationally transmitted. Namely, some research has been specifically devoted to study the effects of the pre-reproductive exposure of mothers to EE on the offspring’s BDNF brain levels (Caporali et al., 2014; Caporali et al., 2015; Cutuli et al., 2015). Interestingly, while the pre-reproductive maternal EE does not modify pups’ brain BDNF protein levels at birth, changes are evident at weaning and in adulthood (Caporali et al., 2014; Cutuli et al., 2015). It may be hypothesized that eventual latent genetic and epigenetic effects of pre-reproductive maternal EE on BDNF signaling need the interaction with the mother and the external ambient (that occurs only after the birth) to be manifest in term of BDNF protein differences. In fact, stable effects of pre-reproductive maternal EE are evident in pups’ brain BDNF levels from weaning onward, and are different in association with the different experiences the pups are exposed to. When BDNF brain expression is evaluated at weaning in pups exposed to repeated motor challenging and exercise, BDNF increases in enriched mother’s offspring at cerebellar and striatal levels, demonstrating an enhanced plasticity expression in the areas involved in motor performance (Caporali et al., 2014). On the other hand, when BDNF expression is evaluated in pups exposed only to maternal care and cage interaction with brotherhood (and not to behavioral testing), BDNF increases at weaning and in adulthood only in hippocampus, the brain area involved in memory formation, and thus solicited by every experience (Cutuli et al., 2015). The exposure after birth to the care of an enriched mother that shows in turn increased brain BDNF level (Caporali et al., 2015; Cutuli et al., 2015) is fundamental for any BDNF change. In fact, the overt increase in hippocampal BDNF levels is not anymore found when the pups are born to an enriched mother but raised until weaning by a standard-reared mother (Caporali et al., 2015).

In line with our results, in a different model of maternal EE, pups born to mothers enriched during pregnancy and lactation do not show variations in cortical or hippocampal BDNF levels few days after birth (Durán-Carabali et al., 2018). In addition, it has been demonstrated that even a specific component of pre-reproductive EE, such as exercise, is able to change offspring’s BDNF brain levels. Parnpiansil et al. (2003) reported that rat pups born to mother exposed to motor enrichment during pregnancy show BDNF mRNA hippocampal expression enhanced at birth, unchanged during lactation and reduced after weaning. More recently, it has been showed that motor enrichment during pregnancy induces enhanced BDNF protein expression in the hippocampus of pups when adult (Gomes Da Silva et al., 2016). Similarly, Park et al. (2013) showed that mice pups born to mother exposed to motor enrichment during pregnancy display enhanced hippocampal BDNF expression after weaning. Furthermore, maternal motor enrichment during pregnancy induces increased BDNF mRNA hippocampal expression in rat pups after weaning (Lee et al., 2006).

On the whole, the available evidence supports the influence of the maternal enrichment during pre-reproductive and post-reproductive periods on the pup brain BDNF-mediated neuroplasticity, regardless of the specific characteristics of the enrichment paradigm.

## Impact of Differently Timed Parental Enrichment on the Oxytocinergic System

The neuropeptide oxytocin is widely implicated in the social behavior of mammalian by modulating maternal care, infant behavior, social bonding, agonistic behavior and social recognition (Lim and Young, 2006; Bosch, 2013; Crespi, 2016). It is synthesized in the magnocellular neurons of the hypothalamic paraventricular nucleus (PVN) and supraoptic nucleus (SON), and is transported along their axons to the posterior pituitary and released from there into the blood stream to act on target organs in the periphery (Veenema, 2009). It is also released in the forebrain and hindbrain regions (Veenema, 2009). Oxytocin receptors are expressed in many brain regions, including cortical, limbic, hypothalamic and brain stem areas (Lee et al., 2009; Veenema, 2009).

Unfortunately, there is a scant literature on the effects of the enrichment on oxytocinergic system either in the dams and offspring. With regards to differently enriched dams, it seems that a potentiation of the oxytocinergic system is linked to increased maternal care. For example, communally reared dams exhibit elevated levels of *post-partum* care and of oxytocin receptor density in the lateral septum across generations (Curley et al., 2009). Similarly, post-weaning enrichment enhances LG behavior and oxytocin receptor binding of low LG offspring in the PVN, medial preoptic area and bed nucleus of the stria terminalis in comparison to low LG offspring housed in standard conditions (Champagne and Meaney, 2007).

A recent study has demonstrated the impact of pre-reproductive maternal EE on the hypothalamic oxytocinergic neurons on mothers and pups (Cutuli et al., 2018). Indeed, enriched dams show an increased number of oxytocinergic neurons either in PVN and SON associated with increased crouching levels and faster pups’ retrieval. As for the adolescent offspring, while no differences have been found in the female pups, the male pups of pre-reproductively enriched dams exhibited higher levels of oxytocinergic neurons in SON and reduced play behavior. Interestingly, the anti-aggressive properties of the oxytocin are well-documented in humans and rodents (Todeschin et al., 2009; Crespi, 2016; Hathaway et al., 2016), and SON is the hypothalamic nucleus selectively activated after the display of offense (Kollack-Walker et al., 1997). Thus, being the social play a sort of preparation for the adult aggressive behavior (Aldis, 1975), the reduction in play behavior observed in male pups of enriched dams may be attributed to reduced aggressive tendencies linked to increased oxytocin levels in SON.

Due to the consistent interactions between oxytocinergic and dopaminergic systems in the establishment and maintenance of mother-pup relationship (Crespi, 2016), the differently enriched dams could be more attracted by pups because of a more rewarding effect of nurturing behaviors. And in turn, their pups could be more soliciting. To our knowledge, only one study has described an increased motivation of male pups born to pre-reproductively enriched females to contact the odor of their mothers (Cutuli et al., 2015). Furthermore, the increased maternal care found in enriched dams may potentiate the oxytocinergic system of their pups through epigenetic modifications, as demonstrated in previous studies (Champagne, 2008).

In conclusion, many questions remain to be answered and further studies are expected to clarify the role of oxytocinergic system in mediating the impact of parental enrichment in the subsequent generations.

## Discussion

An increasing body of evidence from animal studies shows the outcomes of maternal enrichment exposure during, before and/or after the gestation on development, behavior and physiological functioning of the progeny (Arai and Feig, 2011; Taouk and Schulkin, 2016).

In the present review article, we focused on the effects of pre-reproductive exposure of parents to highly stimulating environments on maternal behavior and offspring’s phenotype.

A problem encountered in screening the current (still limited) literature is the diversity of paradigms as for timing of exposure and enhanced stimulations used. Nevertheless, a common pattern emerges in findings. As for dams, the pre-reproductive enrichment tendentially results in increased maternal care, brain BDNF and oxytocinergic levels. As for offspring, the pre-reproductive parental enrichment appears to accelerate the acquisition of complex motor abilities, potentiate cognitive performances and coping skills, reduce anxiety, increase brain BDNF levels and modulate social behavior and oxytocinergic system in a sex-dependent manner.

## Conclusion

On the whole, the available evidence supports an influence of pre-reproductive parental enrichment on mother’s and pups’ brain and behavior. However, given the diversity in the different parental enrichment paradigms, systematic analyses on the selective exposure to EE (and its social, cognitive, physical and motor components) in the different pre-reproductive and post-reproductive periods are required to provide exhaustive evidence on brain and behavior changes induced in pups by parental experiences.

It is interesting to speculate on the mechanisms of inheritance involved in the transmission of the environmental influence from parents to the offspring. The inheritance across generations may involve epigenetic modifications in the germline or can be passed to the offspring through maternal care during early post-natal life (Champagne and Curley, 2009; Bohacek and Mansuy, 2015; Kundakovic and Champagne, 2015). Non-genetic marks fluctuate throughout lifetime and carry important information about previous experiences and faced environments, and their outcomes on the organism (Campos et al., 2014). For these reasons, exposure to enriching experiences may be regarded as therapeutic intervention to support healthy aging (Ambeskovic et al., 2017) or reverse stress detrimental effects (Leshem and Schulkin, 2012).

Finally, the study of the inter- and transgenerational origin of age- or stress-related diseases offers the opportunity to identify predictive or diagnostic biomarkers crucial to develop new interventions according to a tailored clinical approach that claims healthy aging and mental health in today’s population and future generations.

## Author Contributions

DC and LP coordinated the work, wrote, and edited the article. EB, FG, PC, and DL contributed in writing the article.

## Conflict of Interest Statement

The authors declare that the research was conducted in the absence of any commercial or financial relationships that could be construed as a potential conflict of interest.
